# Long-term oral meclozine administration improves survival rate and spinal canal stenosis during postnatal growth in a mouse model of achondroplasia in both sexes

**DOI:** 10.1093/jbmrpl/ziae018

**Published:** 2024-02-24

**Authors:** Hiroto Funahashi, Masaki Matsushita, Ryusaku Esaki, Kenichi Mishima, Bisei Ohkawara, Yasunari Kamiya, Yasuhiko Takegami, Kinji Ohno, Hiroshi Kitoh, Shiro Imagama

**Affiliations:** Department of Orthopaedic Surgery, Nagoya University Graduates School of Medicine, Nagoya, Aichi 466-8550, Japan; Department of Orthopaedic Surgery, Nagoya University Graduates School of Medicine, Nagoya, Aichi 466-8550, Japan; Department of Orthopaedic Surgery, Nagoya University Graduates School of Medicine, Nagoya, Aichi 466-8550, Japan; Division of Neurogenetics, Center for Neurological Diseases and Cancer, Nagoya University Graduate School of Medicine, Nagoya, Aichi 466-8550, Japan; Department of Orthopaedic Surgery, Nagoya University Graduates School of Medicine, Nagoya, Aichi 466-8550, Japan; Division of Neurogenetics, Center for Neurological Diseases and Cancer, Nagoya University Graduate School of Medicine, Nagoya, Aichi 466-8550, Japan; Department of Orthopaedic Surgery, Nagoya University Graduates School of Medicine, Nagoya, Aichi 466-8550, Japan; Department of Orthopaedic Surgery, Nagoya University Graduates School of Medicine, Nagoya, Aichi 466-8550, Japan; Division of Neurogenetics, Center for Neurological Diseases and Cancer, Nagoya University Graduate School of Medicine, Nagoya, Aichi 466-8550, Japan; Department of Orthopaedic Surgery, Aichi Children’s Health and Medical Center, Obu, Aichi 474-8017, Japan; Department of Comprehensive Pediatric Medicine, Nagoya University Graduate School of Medicine, Nagoya, Aichi 466-8550, Japan; Department of Orthopaedic Surgery, Nagoya University Graduates School of Medicine, Nagoya, Aichi 466-8550, Japan

**Keywords:** achondroplasia, meclozine, paralysis, bone growth, spinal canal stenosis

## Abstract

Achondroplasia (ACH) is a skeletal dysplasia characterized by short-limbed short stature caused by the gain-of-function mutations in the fibroblast growth factor receptor 3 (FGFR3) gene. Activated FGFR3, which is a negative regulator of bone elongation, impairs the growth of long bones and the spinal arch by inhibiting chondrocyte proliferation and differentiation. Most patients with ACH have spinal canal stenosis in addition to short stature. Meclozine has been found to inhibit FGFR3 via drug repurposing. A 10-d treatment with meclozine promoted long-bone growth in a mouse model of ACH (*Fgfr3*^ach^ mice). This study aimed to evaluate the effects of long-term meclozine administration on promoting bone growth and the spinal canal in *Fgfr3*^ach^ mice. Meclozine (2 mg/kg/d) was orally administered to *Fgfr3*^ach^ mice for 5 d per wk from the age of 7 d to 56 d. Meclozine (2 mg/kg/d) significantly reduced the rate of death or paralysis and improved the length of the body, cranium, and long bones in male and female *Fgfr3*^ach^ mice. Micro-computed tomography analysis revealed that meclozine ameliorated kyphotic deformities and trabecular parameters, including BMD, bone volume/tissue volume, trabecular thickness, and trabecular number at distal femur of *Fgfr3*^ach^ mice in both sexes. Histological analyses revealed that the hypertrophic zone in the growth plate was restored in *Fgfr3*^ach^ mice following meclozine treatment, suggesting upregulation of endochondral ossification. Skeletal preparations demonstrated that meclozine restored the spinal canal diameter in *Fgfr3*^ach^ mice in addition to improving the length of each bone. The 2 mg/kg/d dose of meclozine reduced the rate of spinal paralysis caused by spinal canal stenosis, maintained the growth plate structure, and recovered the bone quality and growth of axial and appendicular skeletons of *Fgfr3*^ach^ mice in both sexes. Long-term meclozine administration has the potential to ameliorate spinal paralysis and bone growth in patients with ACH.

## Introduction

Achondroplasia (ACH) is a common skeletal dysplasia associated with short-limbed short stature caused by the gain-of-function mutations in the fibroblast growth factor receptor 3 (FGFR3) gene and characterized by relative macrocephaly with frontal bossing, midface hypoplasia, and thoracolumbar kyphosis.^1-4^ Adult patients with ACH often experience spinal canal stenosis, which can progress to lower extremity paralysis and loss of bladder and bowel control. A population-based study in Norway reported that 68% of patients with ACH had neurological symptomatic spinal stenosis.^5^ Furthermore, spinal canal stenosis often develops at a relatively young age after puberty and requires surgery.^5^ Limb lengthening is a treatment option for short-stature ACH in some countries; however, this treatment requires a long treatment period with significant efforts and is associated with many complications.^6^^,^^7^ The magnitude of bone extension has been reported to be a risk factor for bone extension fractures.^8^

Conversely, activated FGFR3 signaling is related to attenuated chondrocyte proliferation and differentiation in the growth plate via signaling pathways, including MAPK pathways.^9^ Areas of the proliferating and hypertrophic zones were found to be narrowed according to the analysis of the growth plate in an ACH mouse model.^10^ Several therapeutic approaches have been developed to target FGFR3 signaling. To date, vosoritide (BMN-111), a C-type natriuretic peptide (CNP) analog,^11^^,^^12^ has been marketed for the treatment of ACH.^13^^,^^14^ We previously demonstrated that meclozine, an anti-motion sickness agent, inhibits the MAPK pathway in FGFR3-activated chondrocytes.^15^ In a mouse model of ACH, oral administration of 1 and 2 mg/kg/d meclozine promotes long-bone growth in a dose-dependent manner, whereas 20 mg/kg/d meclozine is ineffective.^16^ However, these meclozine doses were administered for only 10 d, and the effect of meclozine on spinal canal stenosis remains unclear. Therefore, the long-term administration of meclozine in a mouse model requires its validation in children with ACH.

This study aimed to evaluate the effects of long-term meclozine administration on the onset of paralysis caused by spinal canal stenosis and the enhancement of bone growth in a mouse model of ACH.

## Materials and methods

### Mice

All animal experiments were performed in accordance with the protocols approved by the Animal Care and Use Committee of our institute. The mice were provided with water and standard commercial diet ad libitum and were housed under a 12-h light–dark cycle. All mice were housed in the same racks to ensure that the environment did not change. We used wild-type and *Fgfr3*^ach^ mice (FVB background) expressing a constitutively active FGFR3 mutant carrying p.G380R, observed in patients with ACH under the control of the Col2a1 promoter and enhancer sequences.^17^*Fgfr3*^ach^ mice were kindly provided by Dr David M. Ornitz of Washington University.

### Protocol of meclozine administration

We administered meclozine (MP Biomedicals) to wild-type and *Fgfr3*^ach^ mice. A concentration of 2 mg/kg/d of meclozine was prepared daily by mixing 0.2 mg of meclozine in 1 mL of 0.5% methylcellulose (MC) (4000 centipoise; Sigma-Aldrich), in accordance with our previous study.^16^ Meclozine (2 mg/kg/d) was administered using a feeding tube twice daily for 5 d per wk from the age of 7 d to 56 d (Supplementary Figure S1A). As a control, 10 mL/kg/d of 0.5% MC was administered to untreated mice. *Fgfr3*^ach^ and wild-type mice were randomly divided into meclozine-treated and untreated groups. Mice were euthanized on developing paralysis. Mice were reared simultaneously in the same litter without distinction between meclozine-treated and untreated groups. Males and females were separated to avoid pregnancy when the mice were weaned. The sample size was calculated in accordance with a previous study,^18^ and it was planned to have at least 10 samples per group for each sex (survival rate in treated group, 88%; survival rate in untreated group, 37%; α error, 0.05; power, 0.80). We measured body length on days 0, 7, 14, 21, 28, 35, 42, and 49 after meclozine administration and compared the body length gained between the untreated and meclozine-treated groups. Mice that died or were paralyzed during treatment were excluded when measuring body length. All surviving mice at the end of treatment were subjected to radiographic and histological analyses, and skeletal preparation. To evaluate the histology of spheno-occipital synchondrosis (SOS), meclozine (2 mg/kg/d) was administered to male *Fgfr3*^ach^ and wild-type mice from the age of 7 d to 28 d (Supplementary Figure S1B).

Wild-type mice (2, 20, 50, and 100 mg/kg/d) were treated from the age of 7 d for 10 d to evaluate body weight, liver weight, and liver histology (Supplementary Figure S1C). In addition, 4 and 8 mg/kg/d meclozine were administered to *Fgfr3*^ach^ mice from the age of 7 d for 10 d (Supplementary Figure S1D). To determine the optimal dose of meclozine to promote bone growth in *Fgfr3*^ach^ mice, the same protocol was employed to compare the effects of 1, 2, and 20 mg/kg/d meclozine for 10 d.^16^ We measured body length on days 0, 5, 7, and 10 after meclozine treatment and compared the body length gained between the untreated and meclozine-treated groups. The number of mice used in this study is listed in Supplementary Table S1.

### Radiographic analysis

At the end of the treatment, the mice were subjected to micro-computed tomography (micro-CT) scans (SkyScan1176, Bruker). Three-dimensional (3D) images of the whole body were reconstructed using in-house volume-rendering software,^18^ and individual bone lengths, including the cranium, humerus, radius, ulna, femur, tibia, and foramen magnum, were measured. The sagittal and transverse diameters of the foramen magnum were measured (Supplementary Figure S2A). The kyphotic index^19^ was measured using the scout view obtained during micro-CT (Supplementary Figure S2B). We further evaluated the trabecular bone in the metaphysis of the distal femur using Skyscan NRecon software with 3D algorithms in Skyscan CTAn software according to the manufacturer’s instructions. To assess the trabecular bone parameters of metaphysis, a ROI of 200-μm width was selected, which was 200 μm from the most proximal site of the growth plate (Supplementary Figure S2C). The bone volume/tissue volume (BV/TV), trabecular thickness (Tb.Th), trabecular number (Tb.N), and trabecular separation (Tb.Sp) were calculated as indices of the metaphyseal trabecular compartments. To calculate the BMD at the same sites as the BV/TV measurements, calibration of the Skycan CT system was performed against 0.25 g/cm^3^ and 0.75 g/cm^3^ hydroxyapatite phantoms.

### Histological analysis

Liver weight was measured immediately after sacrifice at the end of the treatment. The livers were fixed in 4% paraformaldehyde at room temperature for 24 h. After embedding in paraffin, samples were cut and stained with H&E. Five images were captured for each sample at 400× magnification using a BZ-X710 microscope (Keyence). We averaged the total number of nuclei from 5 images in each sample.

The femurs were fixed in 4% paraformaldehyde at room temperature for 24 h. After washing with phosphate-buffered saline, the samples were decalcified in 10% EDTA solution at 4°C for 3 wk and then embedded in paraffin. Thin coronal sections (3 μm) were cut and stained with HE and type X collagen (Col X) antibodies (Abcam, Cambridge 14-9771-37). Using the same methods, cranial bases were also embedded in paraffin. Thin sagittal sections (3 μm) were cut at the median of the cranial base and stained with Alcian blue. Images were captured using a BZ-X800 (Keyence) microscope (Keyence). The growth plate and hypertrophic zone areas of the distal femur growth plate were quantified using a BZ-X800 analyzer, based on 200× magnified images.

### Skeletal preparations

After the whole skeleton was harvested from all mice treated with long-term administration of 2 mg/kg/d of meclozine, the specimens were stored in 90% ethanol for 7 d and transferred to 100% acetone for 7 d. Thereafter, the specimens were stained with Alizarin red S (Sigma-Aldrich) and Alcian blue (Sigma-Aldrich) dissolved into glacial acetic acid and ethanol for 3 d at 37°C. The skeletal samples were incubated with 1% potassium hydroxide (KOH) treatment at room temperature for 1 d. KOH concentration was gradually decreased every 2 wk to prevent the bone and cartilage from dissolving. After the soft tissue almost disappeared, the specimens were stored in glycerol. The cranial base, vertebrae (C1, C7, and T13), upper limbs, thorax, and innominate bones were examined under the microscope. To evaluate the space in the spinal canal, the sagittal and transverse diameters of the spinal canal were measured at C1, C7, and T13 (Supplementary Figure S2D).^20^ We measured the SOS width and SOS-the most anterior point of the foramen magnum (Ba) length (Supplementary Figure S2E).^21^ We also measured the thoracic area (Supplementary Figure S2F) and the length of the sternum,^22^ upper arm, lower arm, and innominate bone (Supplementary Figure S2G). All skeletal preparation measurements were performed using ImageJ software.^23^

### Statistical analysis

Student’s *t*-test was used to compare body weight, liver weight, and nuclear number between untreated wild-type mice and littermate wild-type mice treated with each dose of meclozine. One-way ANOVA followed by Tukey’s post hoc test was used to evaluate each parameter in untreated or meclozine-treated wild-type and *Fgfr3*^ach^ mice. Two-way ANOVA with Tukey’s post hoc test was used to compare the 4 curves of body length gained. The log-rank test was performed by drawing a Kaplan–Meier curve to compare the mortality rates between mice treated with or without meclozine. Statistical significance was set at *P* < .05. All analyses were performed using EZR version 1.41 (64-bit), a graphical user interface for R.^24^

## Results

### Long-term administration of 2 mg/kg/d of meclozine improves survival rate and body length of Fgfr3^ach^ mice

**Figure 1:**
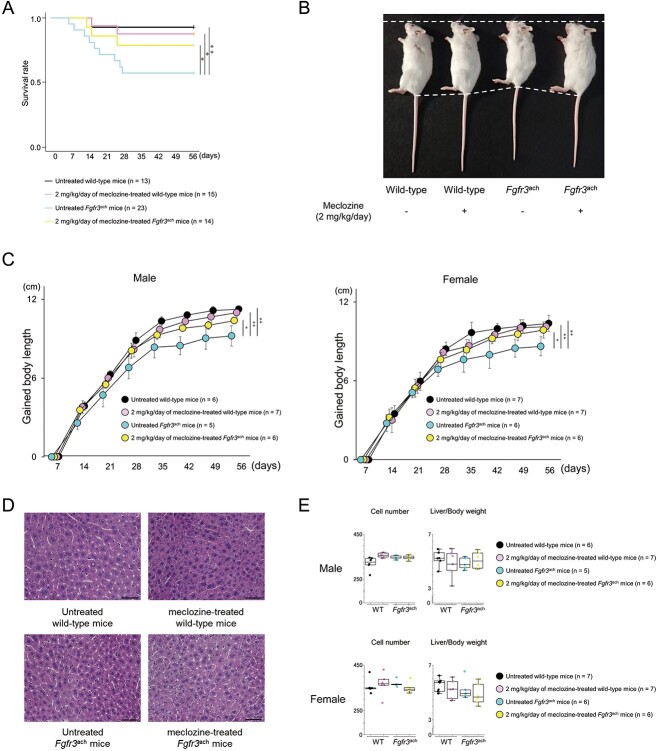
General findings of *Fgfr3*^ach^ mice after 2 mg/kg/d of meclozine administration via the long-term protocol. (A) Survival rate of wild-type and *Fgfr3*^ach^ mice during the treatment of 2 mg/kg/d of meclozine. (B) Representative images captured in a quartetto of 56-d-old littermate female mice. (C) Growth curves of body lengths gained by male and female mice during the treatment with meclozine. (D) Representative histologies captured with 400× magnification of liver sections stained with H&E after the long-term administration of 2 mg/kg/d of meclozine. The scale bar indicates 100 μm. (E) Box plots of the number of hepatocytes in 400× magnification and liver/body weight ratio after the long-term administration of meclozine. Statistical significance was analyzed using log-rank test (A), 2-way ANOVA (C), 1-way ANOVA followed by post hoc Tukey HSD (E). The upper and lower ends of the whiskers and box indicate the maximum and minimum values, and 75th and 25th percentile, respectively. The line and cross point in the box indicated 50th percentile and mean value, respectively. Circular points indicated the values of each sample. Statistical significance was expressed as ^*^P < .05 and ^**^P < .005.

Based on the results of short-term administration from the age of 7 d for 10 d, we found that meclozine doses exceeding 20 mg/kg/d led to weight loss and hepatomegaly (Supplementary Figure S3). The body length peaked at a dose of 2 mg/kg/d of meclozine in *Fgfr3*^ach^ mice (Supplementary Figure 4 and Supplementary Table S2). Therefore, we chose 2 mg/kg/d meclozine for long-term administration from the age of 7 d to 56 d. The survival rate of *Fgfr3*^ach^ mice treated with 2 mg/kg/d of meclozine was significantly lower than that treated without meclozine (untreated *Fgfr3*^ach^ mice vs meclozine-treated *Fgfr3*^ach^ mice: 57.2% vs 78.6%, *P* < .05) (Figure 1A). After excluding mice that died or were paralyzed during the treatment periods, long-term administration of 2 mg/kg/d of meclozine significantly improved the body length in *Fgfr3*^ach^ mice (untreated *Fgfr3*^ach^ mice vs meclozine-treated *Fgfr3*^ach^ mice: male, 9.2 ± 0.8 cm vs 10.4 ± 0.3 cm, *P* < .05; female, 8.6 ± 0.7 cm vs 9.9 ± 0.5 cm, *P* < .05) (Figure 1B and C), without hepatomegaly (Figure 1D) or significant changes in hepatocyte number and liver weight (Figure 1E). Taken together, these results indicate that long-term administration of meclozine not only rescued the decreased survival rate and body length but also demonstrated no hepatic toxicity in *Fgfr3*^ach^ mice.

### Long-term administration of meclozine ameliorates bone length and trabecular architecture of Fgfr3^ach^ mice

**Figure 2:**
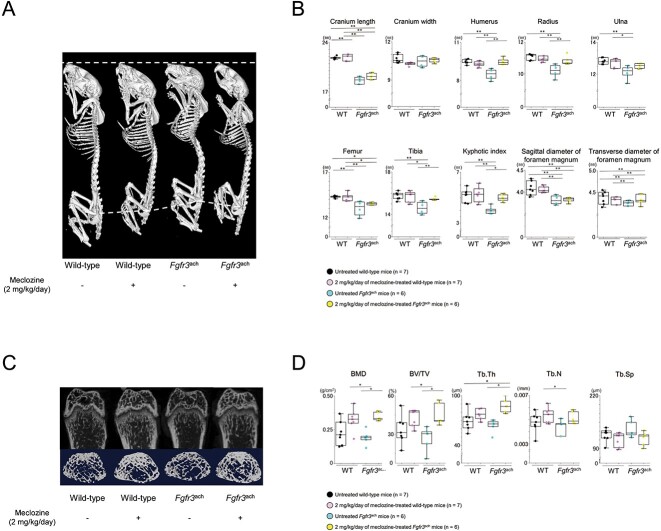
Radiological analyses of *Fgfr3*^ach^ mice after 2 mg/kg/d of meclozine administration via the long-term protocol. (A) Representative 3D micro-CT images captured in a quartetto of 56-d-old female. (B) Box plots of each bone length, KI, and sagittal diameter and transverse diameter of foramen magnum in wild-type and *Fgfr3*^ach^ female mice. (C) Representative micro-CT images of the trabecular bone architecture captured in the quartetto. Upper panels show 2D images of the distal femur, and lower panels show 3D images of metaphyseal trabecular bone. (D) Box plots of the trabecular bone parameters, including BMD, bone volume/total volume (BV/TV), trabecular thickness (Tb.Th), trabecular number (Tb.N), trabecular separation (Tb.Sp), following treatment with 2 mg/kg/d of meclozine in female mice. The upper and lower ends of the whiskers and box in the box-and-whisker diagram indicate the maximum and minimum values, and 75th and 25th percentile, respectively. The line and cross point in the box indicated 50th percentile and mean value, respectively. Circular points indicated the values of each sample. Statistical significance was analyzed using one-way ANOVA, followed by post hoc Tukey HSD. Statistical significance was expressed as ^*^P < .05 and ^**^P < .005.

At the end of the treatment, 3D images reconstructed from micro-CT scans showed that meclozine-treated *Fgfr3*^ach^ mice were larger than untreated *Fgfr3*^ach^ mice (Figure 2A). Quantitative analyses demonstrated that 2 mg/kg/d meclozine rescued the decreased long-bone length, including the humerus, radius, ulna, femur, and tibia (Figure 2B) and restored the downregulation of KI (Figure 2B and Supplementary Figures S5A and S6) and trabecular bone parameters, including BMD, BV/TV, Tb.Th, and Tb.N (Figure 2C and D and Supplementary Figure S5A) in *Fgfr3*^ach^ mice. However, meclozine did not significantly increase the diameter of the foramen magnum in *Fgfr3*^ach^ mice (Figure 2B and Supplementary Figure S5A).

### Meclozine rescues the structure of growth plate in Fgfr3^ach^ mice

**Figure 3:**
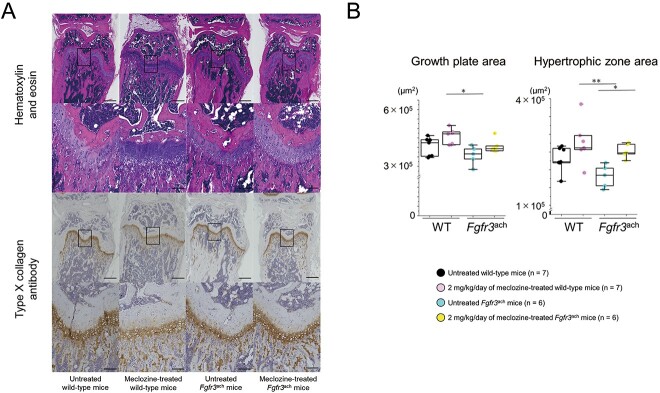
Histology of the growth plate of *Fgfr3*^ach^ mice after 2 mg/kg/d of meclozine administration via the long-term protocol. (A) Representative histological images of growth plate in distal femur at the age of 56 d. The samples were stained with H&E and type X (Col X) collagen antibody, respectively. The scale bars of upper and lower panels in both stainings indicate 500 μm and 100 μm, respectively. (B) Box plots of areas stained with hematoxylin and Col X after the long-term treatment in female mice. The upper and lower ends of the whiskers and box in the box-and-whisker diagram indicate the maximum and minimum values, and 75th and 25th percentile, respectively. The line and cross point in the box indicated the 50th percentile and mean value, respectively. Circular points indicated the values of each sample. Statistical significance was analyzed using one-way ANOVA, followed by post hoc Tukey HSD. Statistical significance was expressed as ^*^P < .05 and ^**^P < .005.

Since the gain-of-function mutation in FGFR3 reduces chondrocyte proliferation and differentiation, only a small number of chondrocytes can mature in the growth plate. Thus, the incomplete hypertrophic zone impairs bone growth in *Fgfr3*^ach^ mice.^10^ To evaluate the effect of meclozine on the promotion of bone growth, we analyzed the growth plate structure. Although the thickness of the growth plate was reduced in *Fgfr3*^ach^ mice, meclozine restored its structure (Figure 3A). Quantitative analyses revealed that 2 mg/kg/d of meclozine significantly improved the area of the hypertrophic zone in the growth plates (Figure 3B and Supplementary Figure S5B).

### Meclozine attenuates spinal canal stenosis in Fgfr3^ach^ mice

**Figure 4:**
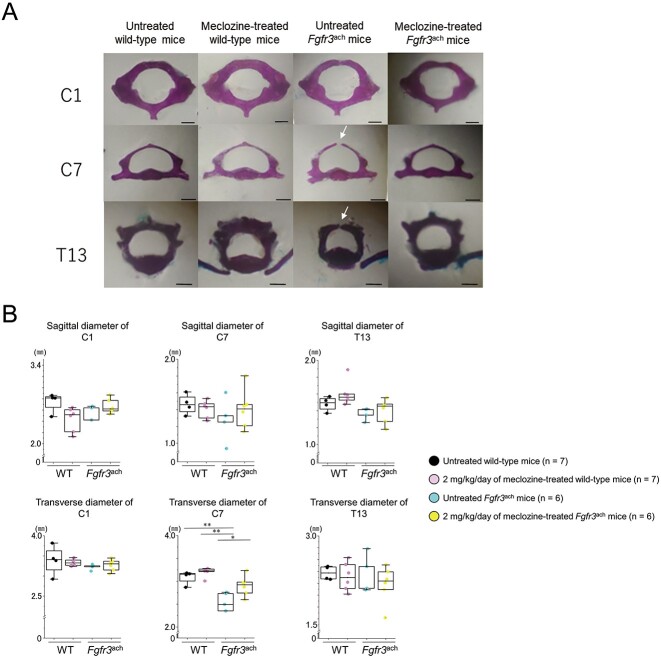
Spinal canal of *Fgfr3*^ach^ mice after 2 mg/kg/d of meclozine administration via the long-term protocol. (A) Representative images of the skeletal preparation of C1, C7, and T13 vertebrae captured in a quartetto of 56-d-old female. White arrows indicate unossified area of the spinal arch. The scale bar indicates 1 mm. (B) Box plots of the sagittal and transverse diameters of the spinal canal in C1, C7, and T13 following the long-term treatment with 2 mg/kg/d of meclozine in female mice. The upper and lower ends of the whiskers and box in the box-and-whisker diagram indicate the maximum and minimum values, and 75th and 25th percentile, respectively. The line and cross point in the box indicated 50th percentile and mean value, respectively. Circular points indicated the values of each sample. Statistical significance was analyzed via one-way ANOVA, followed by post hoc Tukey HSD. Statistical significance was expressed as ^**^P < .005.

Next, we investigated ossification of the spinal arch using skeletal preparations at the age of 56 d. The spinal arches of C1, C7, and T13 were completely ossified and formed tubular structures in untreated and meclozine-treated wild-type mice (Figure 4A). In contrast, some spinal arches were not completely ossified in the untreated *Fgfr3*^ach^ mice (Table 1). Although the spinal arches of C1 were ossified in *Fgfr3*^ach^ mice, 80.0% and 20.0% of C7 and T13 showed incomplete ossification, respectively. Long-term administration of meclozine rescued these immature tubular structures. The diameter of the spinal canal at C7 was significantly recovered by meclozine administration, although there were no significant differences in the diameters of C1 and T13 between untreated and meclozine-treated *Fgfr3*^ach^ mice (Figure 4B and Supplementary Figure S5C).

**Table 1:** Proportions of mice with immature C1, C7, and T13.

	Untreated *Fgfr3*^ach^ mice	Meclozine-treated *Fgfr3*^ach^ mice
Immature C1	0.0%	0.0%
Immature C7	80.0%	33.3%^a^
Immature T13	20.0%	0.0%

a *P* < .05, Untreated *Fgfr3*^ach^ vs meclozine-treated *Fgfr3*^ach^ mice using the chi-squared test

### Administration of 2 mg/kg/d of meclozine rescues the decreased size of axial skeleton and appendicular skeleton in Fgfr3^ach^ mice

**Figure 5:**
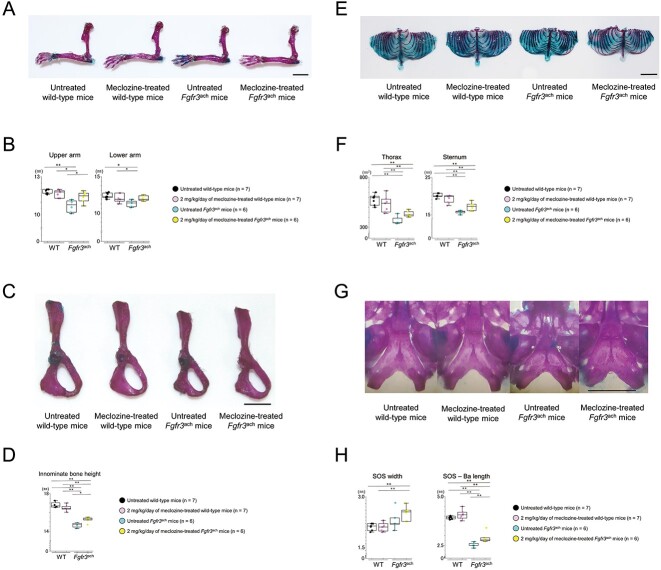
Skeletal preparation of appendicular and axial skeletons of *Fgfr3*^ach^ mice after 2 mg/kg/d of meclozine administration via the long-term protocol. (A) Representative images of skeletal preparation in upper limb after meclozine administration of both mice. The scale bar indicates 5 mm. (B) Box plots of the lengths in upper and lower arms of untreated and meclozine-treated female mice. (C) Skeletal preparation of innominate bone after meclozine treatment. The scale bar indicates 5 mm. (D) Box plots of innominate bone height after the long-term administration of meclozine in female mice. (E) Representative skeletal preparation images of thorax. The scale bar indicates 1 cm. (F) Box plots of thorax area and sternum length after meclozine treatment in female mice. (G) Skeletal preparation of the cranial base. The scale bar indicates 1 cm. (H) Box plots of width and length in cranial base in female mice. SOS and Ba indicate SOS and most anterior point of the foramen magnum, respectively. The upper and lower ends of the whiskers and box in the box-and-whisker diagram indicate the maximum and minimum values, and 75th and 25th percentile, respectively. The line and cross point in the box indicated 50th percentile and mean value, respectively. Circular points indicated the values of each sample. Statistical significance was analyzed using one-way ANOVA, followed by post hoc Tukey HSD. Statistical significance was expressed as ^*^P < .05 and ^**^P < .005.

We evaluated bone length using skeletal preparations in addition to micro-CT scans. We confirmed that meclozine treatment rescued the downregulation of the long-bone length of the upper extremities (Figure 5A and B and Supplementary Figure S5D), the long axis of innominate bone length (Figure 5C and D), the size of the rib cage and sternum (Figure 5E and F and Supplementary Figure S5D), and the axial length of the cranial base (Figure 5G and H and Supplementary Figure S5D) in *Fgfr3*^ach^ mice. Histological analyses of the cranial base revealed that meclozine tended to delay premature closure of SOS in *Fgfr3*^ach^ mice (Supplementary Figure S7).

## Discussion

This study demonstrated that long-term administration of meclozine ameliorated bone growth disturbances without hepatic toxicity in *Fgfr3*^ach^ mice. We found that treatment with meclozine from the age of 7 d reduced the rate of paralysis by maintaining spinal alignment and sufficient space in the spinal canal in parts of the vertebra. Furthermore, meclozine restored the hypertrophic zone in the growth plate and promoted the growth of axial and appendicular skeletons in *Fgfr3*^ach^ mice.

Currently, inhibitors of FGFR3 signaling have been evaluated in each clinical phase.^25^ Only vosoritide (CNP analog),^11^^,^^12^ which inhibits the MAPK pathway via PKG2-mediated inhibition of RAF phosphorylation, has been approved for ACH treatment.^26^ Subcutaneous injection of a CNP analog increases long-bone length in a dose-dependent manner and restores growth plate structure in a mouse model of ACH.^11^ In human clinical trials, a 52-wk treatment with vosoritide was well tolerated and 1.5 cm of average height gained per year was obtained after treatment in pediatric patients with ACH.^13^^,^^14^ Similar to the in vitro and in vivo studies evaluating CNP, meclozine inhibited the MAPK pathway in FGFR3 signaling.^15^

We previously demonstrated that meclozine inhibited ERK and p38 but not JNK phosphorylation in FGFR3-mediated chondrocytes.^27^ In another study using osteoclasts, ERK and p38 phosphorylation were inhibited by meclozine in receptor activator for nuclear factor-kappaB ligand signaling, but JNK phosphorylation was not affected.^28^ These results suggest that meclozine inhibits phosphorylation at the MAPKKK level or upstream in the MAPK pathway. The inhibitory effect of meclozine on the MAPK pathway improved proliferation and differentiation of FGFR3-mediated chondrocytes,^15^ and bone growth in *Fgfr3*^ach^ mice on subjecting 21-d-old mice to a 3-wk treatment^29^ and 7-d-old mice to a 10-d treatment^16^; the current study additionally indicated that meclozine restored the structure of the growth plate. Interestingly, meclozine also ameliorated the abnormal structure of the growth plate and trabecular architecture of the metaphysis in a mouse model of X-linked hypophosphatemia (Hyp mice).^30^ Because the MAPK pathway is upregulated in Hyp mice,^31^ meclozine might attenuate these phenotypes.

In this study, long-term administration of meclozine from the age of 7 d not only extended part of the spinal canal but also reduced incomplete ossification in the spinal arch in *Fgfr3*^ach^ mice. A soluble FGFR3, which is another FGFR3 inhibitor, also reversed the spinal canal stenoses and the unossified spinal arch in *Fgfr3*^ach^ mice.^22^ Since ossifying the spinal arch is developed through endochondral ossification from the ossification centers,^32^ FGFR3 inhibitors may rescue the inhibited endochondral ossification of the spinal arch in *Fgfr3*^ach^ mice. The small foramen magnum of *Fgfr3*^ach^ mice was rescued after crossing with CNP-overexpressing transgenic mice.^33^ However, enhancement of the foramen magnum area in a mouse model of ACH by administration of a CNP or a CNP analog has not been demonstrated. The size of the foramen magnum in a mouse model of ACH was increased by subcutaneous injection of NVP-BGJ398 from the age of 1 d.^34^ Because premature synchondrosis closure of the cranial base and spinal arch has been observed in different mouse models of ACH,^35^^,^^36^ the administration of meclozine should be initiated before the age of 7 d in *Fgfr3*^ach^ mice to enlarge the foramen magnum. Although oral administration to mice before the age of 7 d is technically difficult, meclozine administration at an earlier age would lead to the expansion of the foramen magnum in *Fgfr3*^ach^ mice.

Most FGFR3 signaling inhibitors are injectable. Daily subcutaneous injections are required to administer vosoritide in pediatric patients with ACH. Therefore, oral medication is ideal for long-term administration in children. The intraperitoneal injection of statins promotes bone growth in *Fgfr3*^ach^ mice by attenuating *FGFR3*^37^; however, the safety of statin use in young people has yet to be confirmed. ASP5878, an orally administered pan-specific FGFR inhibitor originally developed as an anticancer drug,^38^ enhances bone growth in *Fgfr3*^ach^ mice.^39^ Further research with ASP5878 is needed before it can be applied in clinical settings. In contrast, meclozine has been used in children for anti-motion sickness for more than 50 yr.^40^ There were no reports of severe adverse events after repeated doses of meclozine (25 mg/d) for 3 mo in patients with developmental dyslexia aged 9–14 yr.^41^ In our previous pharmacokinetic analyses,^16^ the peak drug concentration (C_max_) and area under the concentration-time curve of 2 mg/kg meclozine in mice were lower than those obtained with 25 mg/kg/d in adult humans,^42^ which is the dose used for the treatment of motion sickness. Therefore, long-term administration of 25 mg/kg/d or less of meclozine is likely to be a clinically attainable dose for promoting bone growth in pediatric patients with ACH.

The current study had several limitations. First, age equivalence between mice and humans is unknown. However, meclozine administration for human ACH should be initiated as early as possible after birth to enhance the spinal canals. Second, we did not perform the dissection to determine the specific causes of early death. A previous study indicated that early mortality of *Fgfr3*^ach^ mice seemed to be caused by respiratory failure probably due to the spinal canal stenosis.^22^ Third, we used only wild-type mice to evaluate body weight, liver weight, and histology after the administration of 2, 20, 50, and 100 mg/kg/d of meclozine because a sufficient number of *Fgfr3*^ach^ mice was unavailable. Because 20 mg/kg/d of meclozine did not promote bone growth and the plasma concentration after repeated doses indicated accumulation according to pharmacokinetic analyses,^16^*Fgfr3*^ach^ mice may not need to be used for evaluating higher doses. Fourth, meclozine did not promote skeletal growth similar to the *Fgfr3*-knockout mice. The limited effects of meclozine on promoting skeletal growth might be caused by the delayed timing of starting the administration and the inhibition of limited pathways in FGFR3 signaling. Finally, meclozine is not guaranteed to be safe for long-term administration in children with ACH. Although preclinical safety studies have been completed, including 2-wk repeated-dose toxicity studies, toxicokinetic and PK studies, genotoxicity studies, safety pharmacology and pharmacodynamic studies, and juvenile animal toxicity studies, further safety studies employing long-term administration of meclozine are required until application for approval.

In conclusion, long-term administration of meclozine ameliorated paralysis caused by spinal deformities in growing *Fgfr3*^ach^ mice, in addition to promoting bone length and quality. Meclozine administration at an earlier age could potentially expand the spinal canal in patients with ACH. Further studies are required to improve the foramen magnum stenosis in patients with ACH.

## Supplementary Material

supplemental_clear_ziae018

## Data Availability

The data supporting the findings of this study are available from the corresponding author upon request.
