# Supplementary material for: Long-term oral meclozine administration improves survival rate and spinal canal stenosis during postnatal growth in a mouse model of achondroplasia in both sexes
Source: JBMR Plus. 2024 Feb 24;8(4):ziae018. doi: 10.1093/jbmrpl/ziae018 (PMC10972533; doi:10.1093/jbmrpl/ziae018)
Supplement: supplemental_clear_ziae018 [file supplemental_clear_ziae018.docx]

| Supplementary Table 1. Number of mice used in the different experiments | | | | | | |
| --- | --- | --- | --- | --- | --- | --- |
| Treatment duration | Meclozine dose administered | Number of mice | | | | |
|  |  | Wild-type | |  | *Fgfr3*^ach^ | |
|  |  | Vehicle | Meclozine |  | Vehicle | Meclozine |
| From 7 days to 56 days | 2 mg/kg/day | 13 | 15 |  | 24 | 13 |
| From 7 days to 28 days | 2 mg/kg/day | 4 | 4 |  | 4 | 4 |
| From 7 days to 17 days | 2, 20, 50, and 100 mg/kg/day | 13 | 15 |  | - | - |
| From 7 days to 17 days | 4 and 8 mg/kg/day | 23 | - |  | 13 | 14 |
| Total |  | 87 | |  | 72 | |

| Supplementary Table 2. Relative body length after administration of 1, 2, 4, and 8 mg/kg/day meclozine from the age of 7 days for 10 days | | | | |
| --- | --- | --- | --- | --- |
| Meclozine (mg/kg/day) | Number of mice | Relative body length (%) | P value | Study |
| 1 | 7 | 103.0 | 0.69 | Previous study  (16) |
| 2 | 5 | 106.7 | < 0.05 |  |
| 4 | 7 | 105.8 | < 0.05 | Current study |
| 8 | 7 | 99.2 | 0.74 |  |


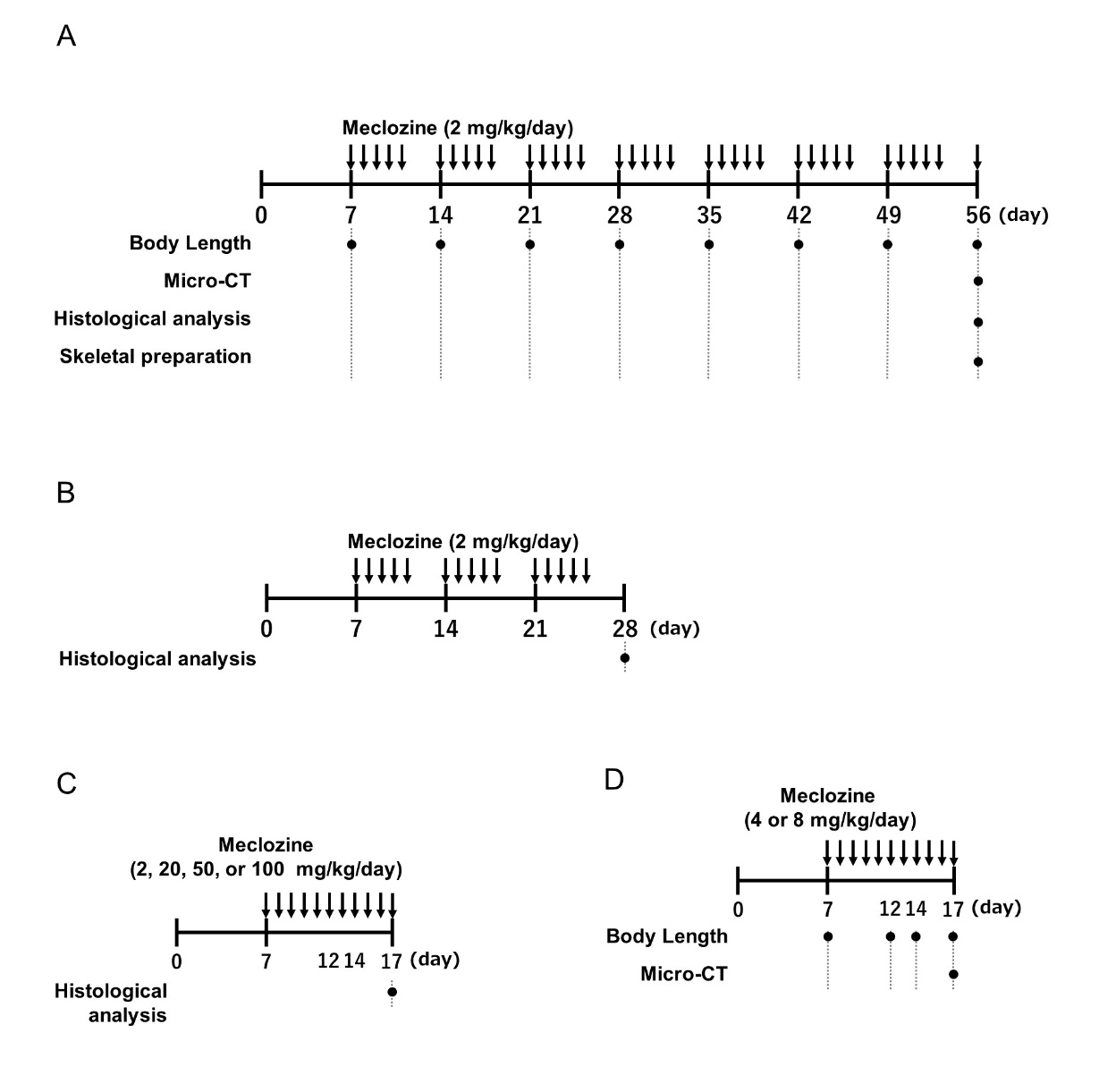


Supplemental Fig. 1.

Protocol for the administration of meclozine to mice. (A) Protocol for long-term (49 days) administration of meclozine (2 mg/kg/day) to wild-type and *Fgfr3*^ach^ mice. Body length was measured every 7 days from the age of 7 days. At the end of treatment, micro-computed tomography (micro-CT), histological analysis, and skeletal preparation were performed. (B) Protocol for the 21-day administration of mecoizine (2 mg/kg/day) to wild-type and Fgfr3ach mice. At the end of the treatment, histological analysis of the spheno-occipital synchondrosis (SOS) was performed. (C) Protocol for the 10-day administration of meclozine (2, 20, 50, or 100 mg/kg/day) was employed for wild-type mice. At the end of the treatment, body weight and liver weight were measured, and histological analysis of the liver was performed. (D) Protocol for the 10-day administration of meclozine (4 or 8 mg/kg/day) to wild-type and *Fgfr3*^ach^ mice. Body length was measured at the ages of 7, 12, 14, and 17 days, and micro-CT was performed at the end point.


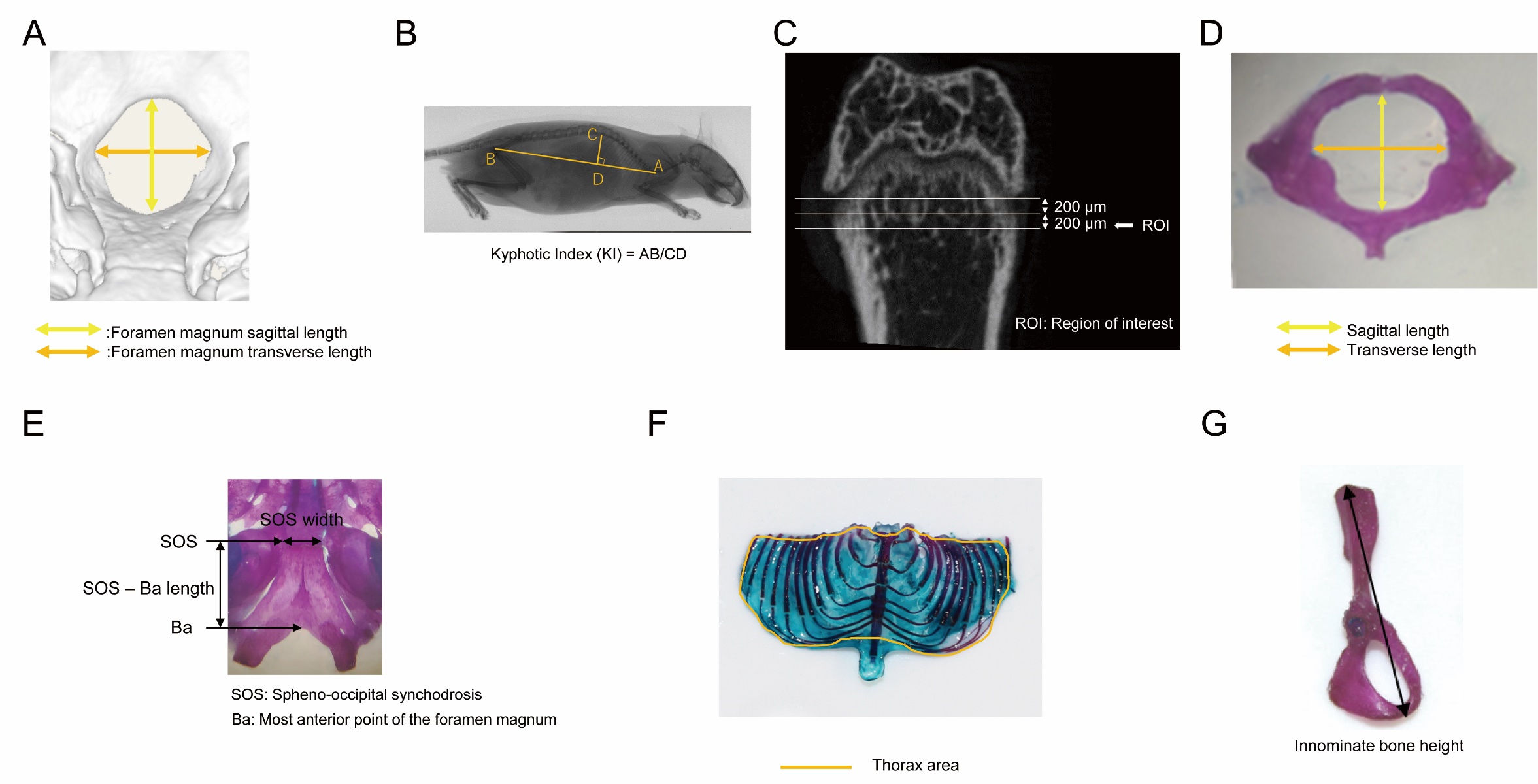


Supplemental Fig. 2.

Measurement Methods. (A) The sagittal and transverse lengths of the foramen magnum were measured using three-dimensional image via micro-computed tomography (micro-CT). (B) The kyphotic index (KI) was measured using AB/CD with a scout view at the time of micro-CT scan. Line AB is drawn between the posterior ends of C7 and L6. Line CD is farthest from line AB. (C) Region of interest (ROI) of trabecular bone parameters was selected as indicated. (D) The sagittal and transverse lengths of the spinal canal were measured using skeletal preparations. (E) SOS width and SOS-Ba length. (F) The thoracic area is measured in the area enclosed by the yellow line. (G) The innominate bone height was measured between the top of the iliac crest and the bottom of the inferior iliac ramus.


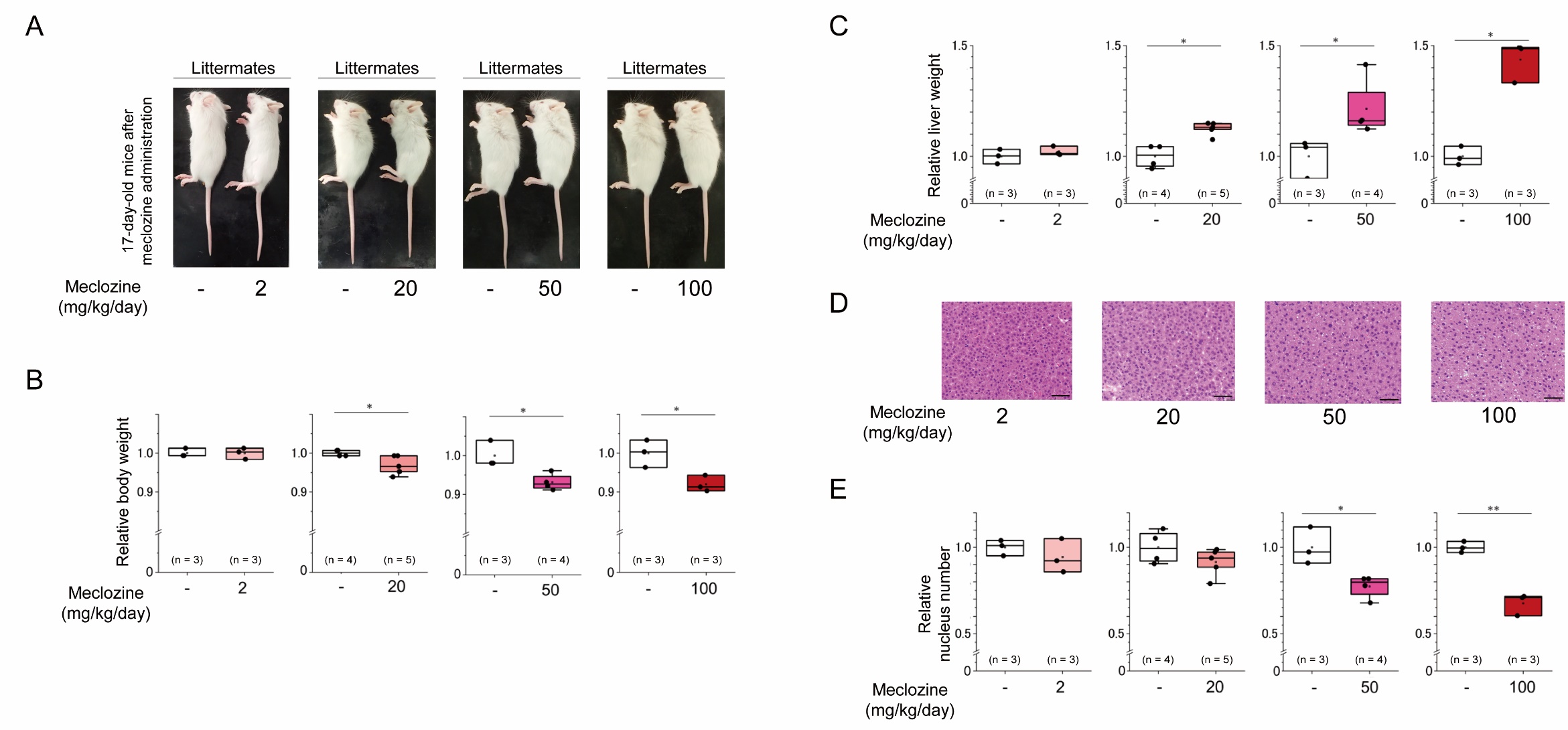


Supplemental Fig. 3.

Body weight and liver findings of wild-type mice after treatment with 2, 20, 50, and 100 mg/kg/day of meclozine using the short-term protocol. (A) Representative images of 17-day-old littermates of wild-type mice after 7-day treatment with 2, 20, 50, and 100 mg/kg/day meclozine. (B, C) Relative body weight (B) and liver weight (C) after each dose of meclozine compared to untreated littermates of wild-type mice. (D) Representative histologies captured with 400× magnification of liver sections stained with hematoxylin and eosin. The scale bar indicates 100 µm. (E) Relative number of nuclei at 400× magnification in liver sections after treatment with each dose of meclozine. The upper and lower ends of the whiskers and box in the box-and-whisker diagram indicate the maximum and minimum values and the 75^th^ and 25^th^ percentiles, respectively. The line and crossing point in the box indicate the 50^th^ percentile and mean value, respectively. The circles indicate the values of each sample. Statistical significance was analyzed using Student’s t-test for each dose administered to meclozine-treated versus untreated mice. Statistical significance is expressed as * p < 0.05, ** p < 0.005.


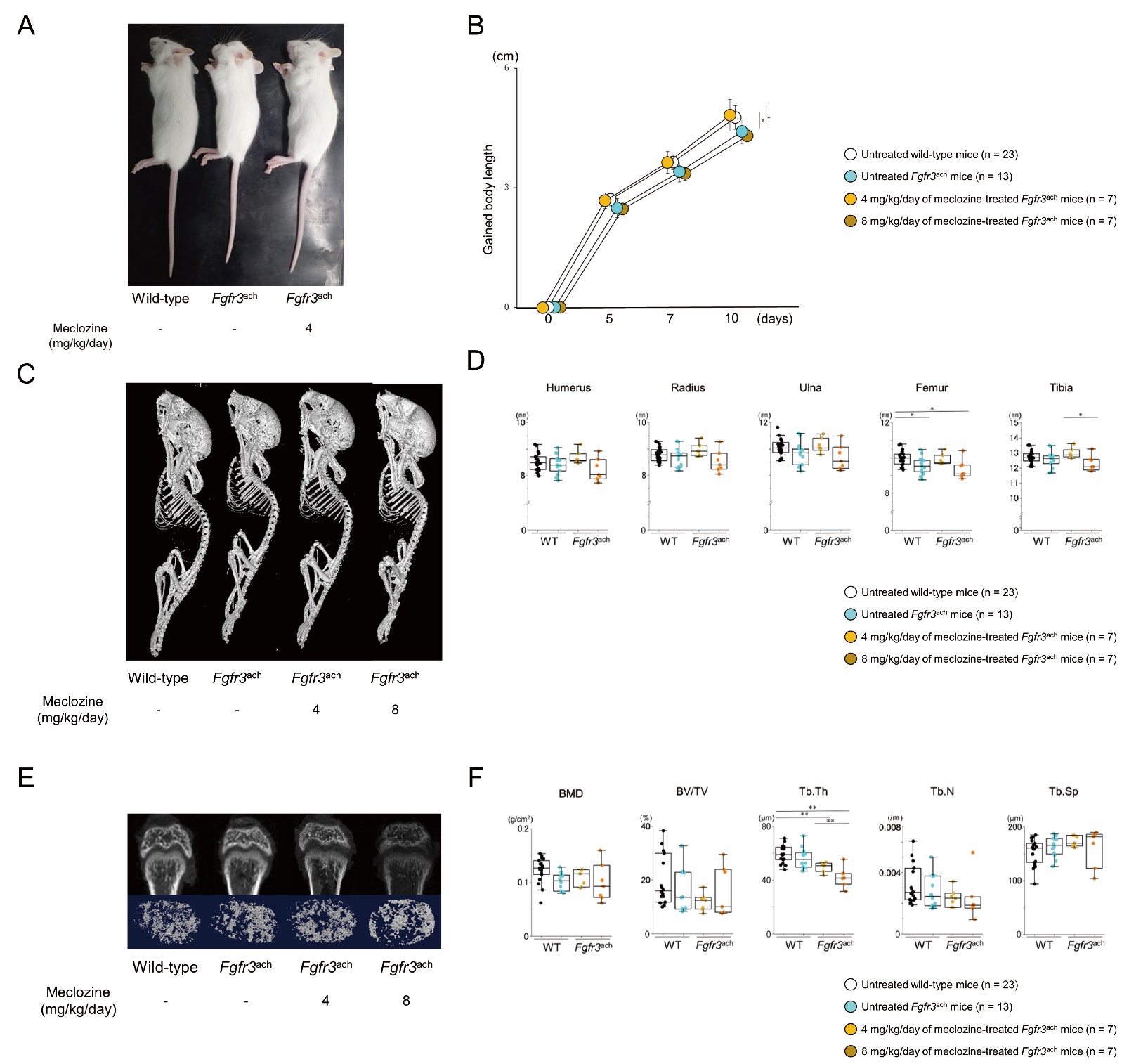


Supplemental Fig. 4.

Effects of 4 and 8 mg/kg/day meclozine on promoting bone elongation in *Fgfr3*^ach^ mice using a short-term protocol. (A) Representative images captured from three 17-day-old female littermates. (B) Growth curve of body length gained during treatment with 4 or 8 mg/kg/day meclozine. (C) Representative reconstructed 3D micro-CT images of 17-day-old mice treated with 4 mg/kg/day or 8 mg/kg/day meclozine. (D) Box plots of each bone length, including the cranium, humerus, radius, ulna, femur, tibia, and vertebrae (L1-5) after treatment. (E) Representative images of trabecular bone architecture in the distal femur reconstructed using micro-CT. The upper and lower panels show 2D and 3D images of the trabecular bone, respectively. (F) Box plots of bone mineral density (BMD), bone volume/total volume (BV/TV), trabecular thickness (Tb.Th), number (Tb.N), and separation (Tb.Sp) after treatment. The upper and lower ends of the whiskers and box in the box-and-whisker diagram indicate the maximum and minimum values and the 75^th^ and 25^th^ percentiles, respectively. The line and crossing point in the box indicate the 50^th^ percentile and mean value, respectively. The circles indicate the values of each sample. Statistical significance was analyzed using one-way ANOVA followed by Tukey’s post-hoc HSD test. Statistical significance is expressed as * p < 0.05, ** p < 0.005.


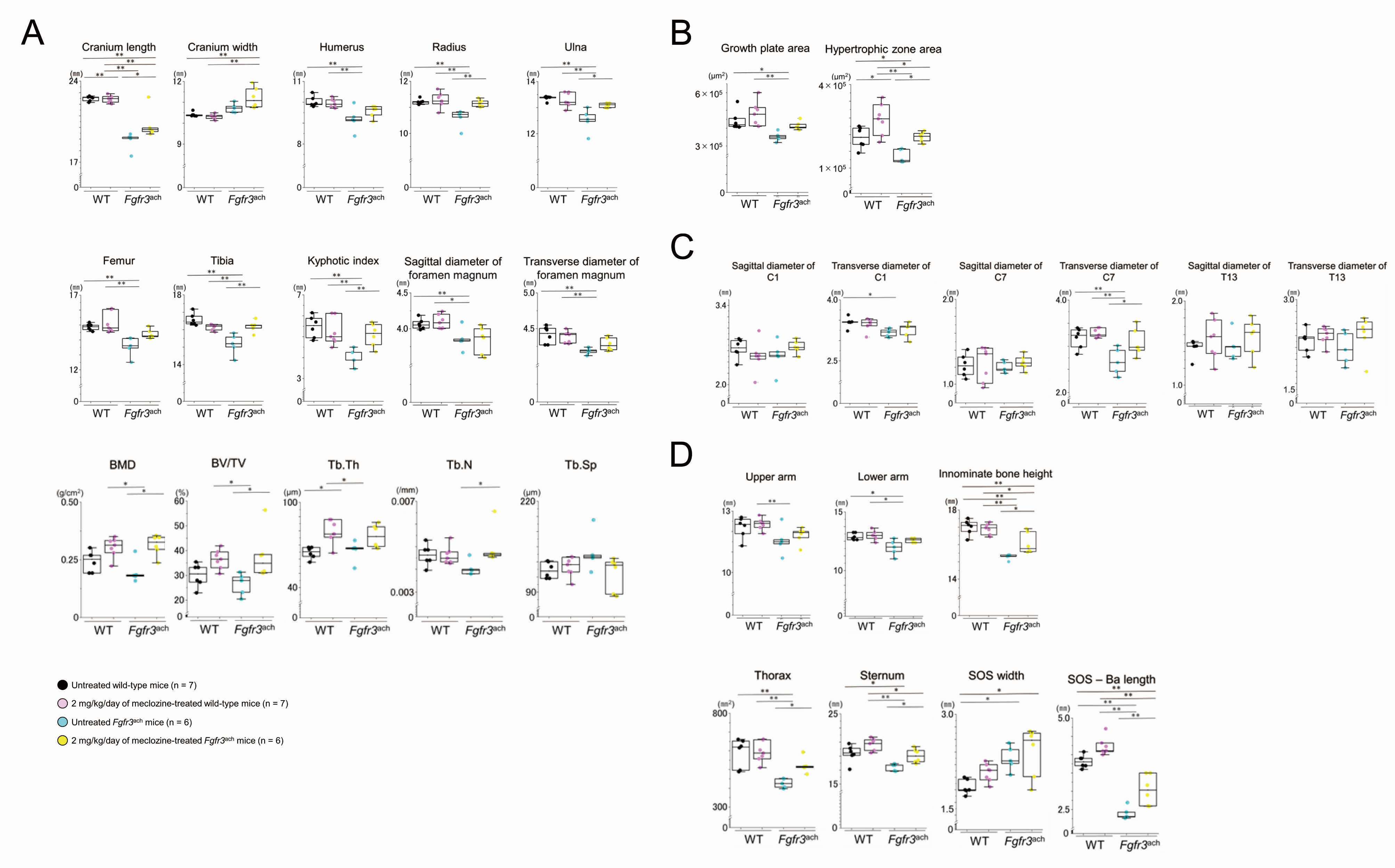


Supplemental Fig. 5.

(A) Radiological analyses of *Fgfr3*^ach^ mice after 2 mg/kg/day of meclozine administration via the long-term protocol. Box plots of each bone length, kyphotic index, and sagittal diameter and transverse diameter of foramen magnum of male wild-type and *Fgfr3*^ach^ mice. (D) Box plots of the trabecular bone parameters, including bone mineral density (BMD), bone volume/total volume (BV/TV), trabecular thickness (Tb.Th), trabecular number (Tb.N), trabecular separation (Tb.Sp), following the treatment with 2 mg/kg/day of meclozine in female mice. (B) Box plots of areas stained with hematoxylin and Col X after the long-term treatment in male mice. (C) Spinal canal of *Fgfr3*^ach^ mice after 2 mg/kg/day of meclozine administration via the long-term protocol. Box plots of the sagittal and transverse diameters of the spinal canal in C1, C7, and T13 following long-term treatment with 2 mg/kg/day of meclozine in female mice. (D) Skeletal preparation of appendicular and axial skeletons of male wild-type and *Fgfr3*^ach^ mice after 2 mg/kg/day of meclozine administration via the long-term protocol. Box plots of the lengths in upper arm, lower arms, innominate bone height, thorax area and sternum length, width in cranial base and length in cranial base. The upper and lower ends of the whiskers and box in the box-and-whisker diagram indicate the maximum and minimum values, and 75th and 25th percentile, respectively. The line and cross point in the box indicated 50th percentile and mean value, respectively. Circle points were indicted values of each sample. Statistical significance was analyzed using one-way ANOVA, followed by post hoc Tukey HSD. Statistical significance is expressed as * p < 0.05 and ** p < 0.005.


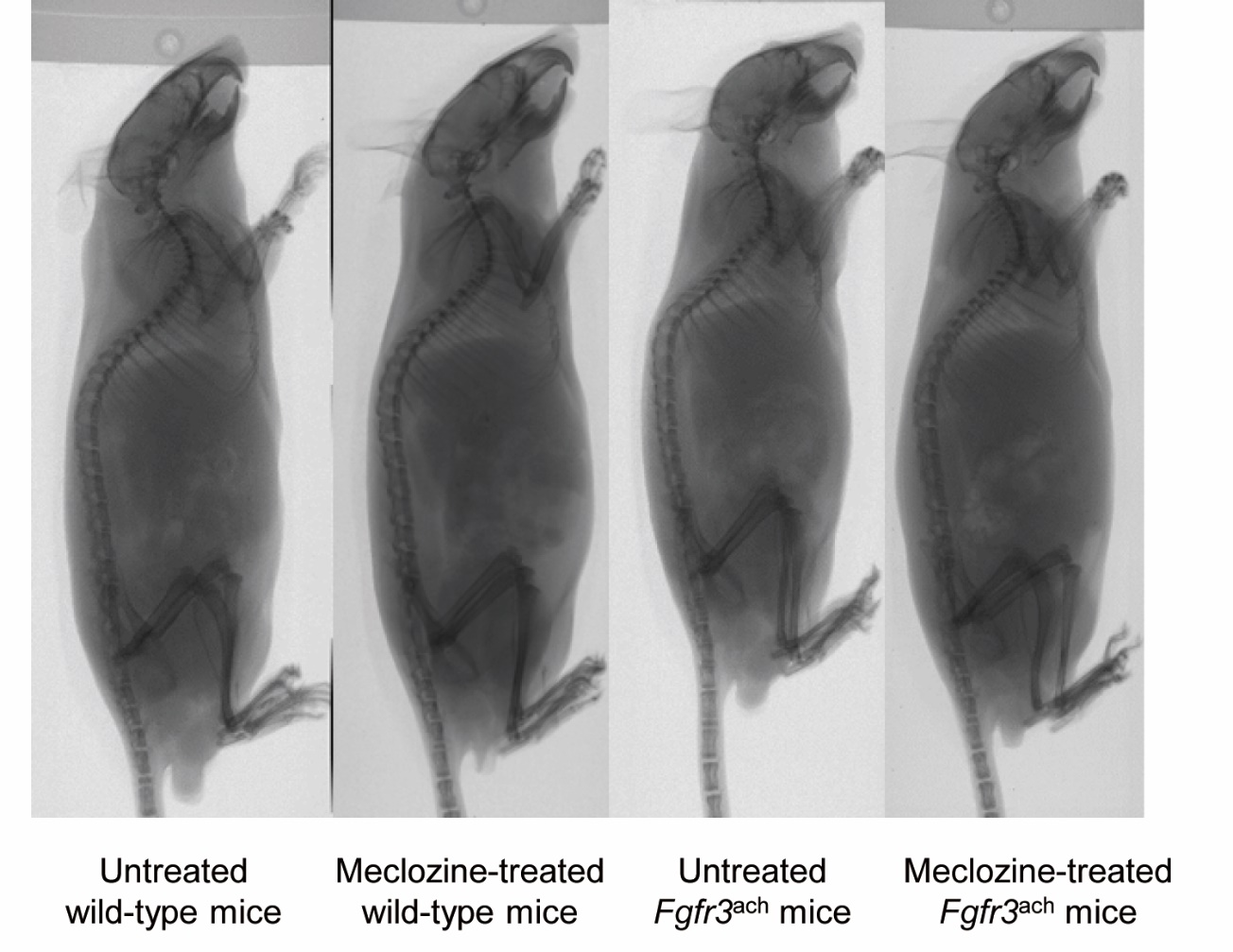


Supplemental Fig. 6.

Representative image of scout view from micro-CT scan showing four littermate female mice at the age of 56 days used to evaluate the kyphotic index (KI).


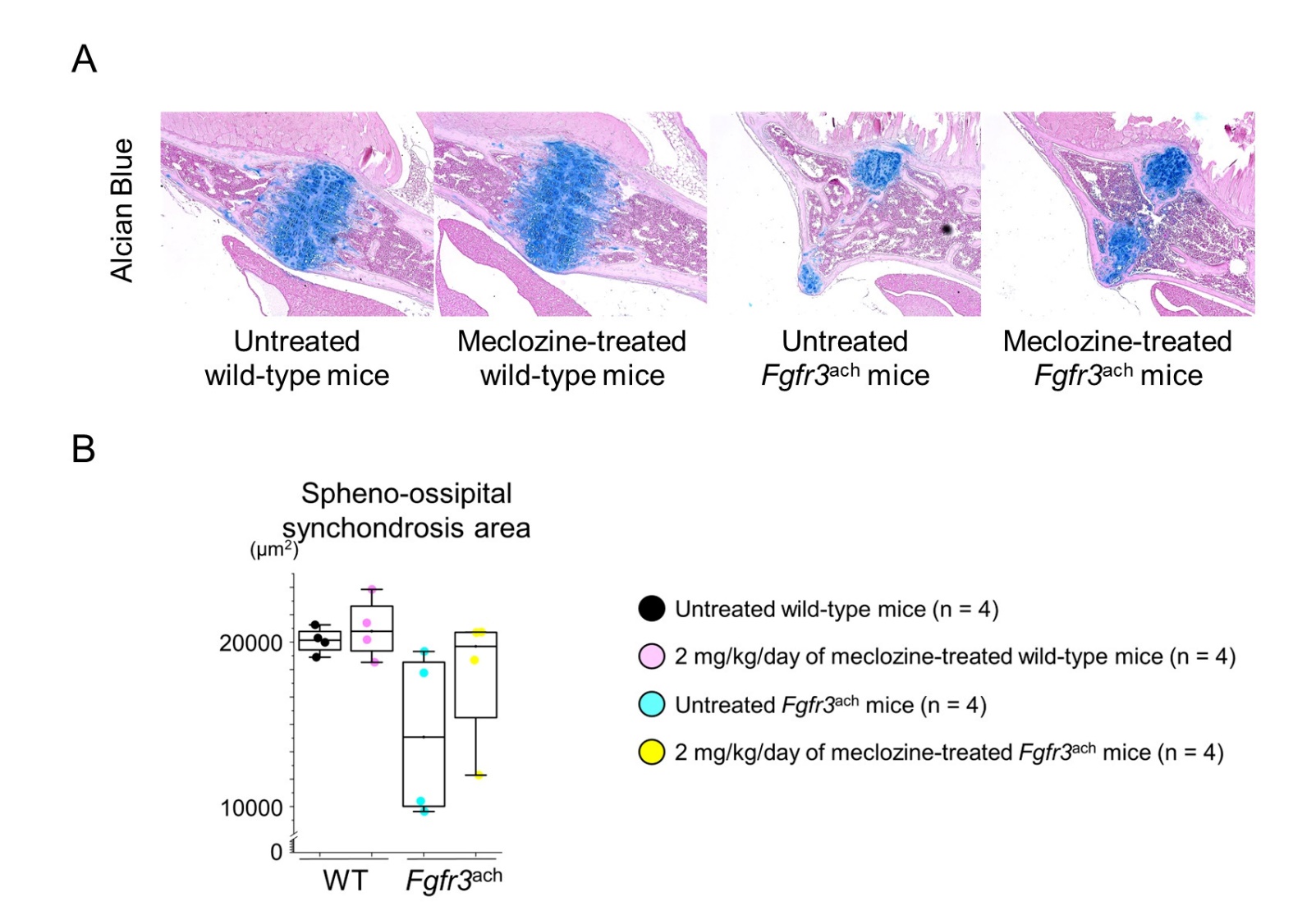


Supplemental Fig. 7.

(A) Histology of the spheno-occipital synchondrosis (SOS) in wild-type and *Fgfr3*^ach^ mice after the 21-day administration of meclozine (2 mg/kg/day). Representative histological images of SOS at the age of 28 days. The samples were stained with alcian blue. The scale bars indicate 200 µm. (B) Box plots of areas stained with alcian blue after the 21-day treatment in male mice. The upper and lower ends of the whiskers and box in the box-and-whisker diagram indicate the maximum and minimum values, and 75^th^ and 25^th^ percentile, respectively. The line and cross point in the box indicated the 50^th^ percentile and mean value, respectively. Circle points were indicted values of each sample. Statistical significance was analyzed using one-way ANOVA, followed by post hoc Tukey HSD.
